# Inference and uncertainty quantification of stochastic gene expression via synthetic models

**DOI:** 10.1098/rsif.2022.0153

**Published:** 2022-07-13

**Authors:** Kaan Öcal, Michael U. Gutmann, Guido Sanguinetti, Ramon Grima

**Affiliations:** ^1^ School of Informatics, University of Edinburgh, Edinburgh EH9 3JH, UK; ^2^ School of Biological Sciences, University of Edinburgh, Edinburgh EH9 3JH, UK; ^3^ Scuola Internazionale Superiore di Studi Avanzati, 34136 Trieste, Italy

**Keywords:** Bayesian inference, uncertainty quantification, chemical master equation, synthetic likelihoods, stochastic modelling

## Abstract

Estimating uncertainty in model predictions is a central task in quantitative biology. Biological models at the single-cell level are intrinsically stochastic and nonlinear, creating formidable challenges for their statistical estimation which inevitably has to rely on approximations that trade accuracy for tractability. Despite intensive interest, a sweet spot in this trade-off has not been found yet. We propose a flexible procedure for uncertainty quantification in a wide class of reaction networks describing stochastic gene expression including those with feedback. The method is based on creating a tractable coarse-graining of the model that is learned from simulations, a *synthetic model*, to approximate the likelihood function. We demonstrate that synthetic models can substantially outperform state-of-the-art approaches on a number of non-trivial systems and datasets, yielding an accurate and computationally viable solution to uncertainty quantification in stochastic models of gene expression.

## 1. Introduction

The stochasticity of biological processes at the single-cell level is one of the major paradigm shifts of twenty-first-century biology [1–3]. Modern experimental methods, ranging from advanced microscopy to single-cell sequencing [4–6], have confirmed and detailed the pervasiveness of stochasticity in cellular biology. While these discoveries open new perspectives on the fundamental functioning of living systems, they also create novel challenges towards the development of mathematical models of biological processes, accentuating the role of statistical inference and uncertainty quantification in any modelling effort.

Biological variability is the result of many concomitant processes. A major source of noise (intrinsic noise) stems from the random timing of chemical reactions and is particularly important for reaction systems involving a small number of molecules of a certain species, as in many gene regulatory systems. The chemical master equation (CME) [7] has been broadly adopted as a general framework to describe the intrinsic stochastic dynamics of chemical reaction networks [8]. While the CME benefits from an elegant mathematical formulation, its exact analytical solution is only known in a few instances [8]; on the other hand, the stochastic simulation algorithm (SSA) [9] provides a Monte Carlo method to perform simulations of systems described by the CME.

Bayesian inference, the gold standard for capturing model and parameter uncertainty, relies on the likelihood function *p*(**x**_obs_|***θ***) to estimate parameters ***θ*** given observations **x**_obs_. For biochemical reaction networks, computing the likelihood requires a closed-form expression for the solution of the CME, which is generally unavailable: while the forward problem of generating samples from the CME can be solved efficiently using the SSA, the backward problem of computing the probability of samples cannot. As a consequence, Bayesian inference for biochemical reaction networks often relies on a variety of approximations to the likelihood function [8,10].

Among the most well known of these are the finite-state projection (FSP) [11], continuum approximations [7,12] and moment equations [13]. The FSP solves the CME on a finite truncation of the state space, whose size typically grows exponentially in the number of species; in practice, this approach relies on computationally intensive approximations [14–16] for more complex systems. Continuum approximations to the CME based on stochastic differential equations, such as the chemical Langevin formalism [12] and the linear noise approximation (LNA) [7] are limited to systems with small noise and in the case of the latter, Gaussian copy number distributions. Moment equations can be derived from the CME and used to construct an approximate likelihood function [17]; for systems with bimolecular reactions, the moment equations have to be ‘closed’ by a process called moment closure, which yields approximate solutions of highly variable quality [13]. These approaches, termed moment-based inference (MBI), are commonly used in practice [17–23] and usually very scalable, but the error introduced by the approximations can be difficult to quantify. Recent work [23–25] has pointed out that these methods can perform poorly for some systems and lead to biased or overly uncertain parameter estimates. We refer to the extensive review [8] for a more thorough exposition of various approximation methods for the CME and their application to parameter inference.

An alternative to analytical approximations of the CME is provided by *simulator-based* inference [26], which relies on simulations of the original model to estimate the likelihood using Monte Carlo methods. This family of methods only requires the ability to perform simulations of the model, which for biochemical reaction networks can be readily obtained using the SSA. Simulator-based approaches are mostly model-agnostic and can be easily adapted to many different problems, but due to their generality they typically require many simulations to produce a fully data-driven approximation of the likelihood.

Perhaps the best-known simulator-based inference method is approximate Bayesian computation (ABC) [27,28]. ABC replaces the likelihood *p*(**x**_obs_|***θ***) with *p*(*d*(**x**_obs_, **x**) ≤ ***ɛ***|***θ***), the probability that the model generates outputs within a tolerance ***ɛ*** of the observed data, where *d*( · , · ) denotes an appropriately chosen discrepancy measure. The posterior is then estimated by repeatedly sampling parameters and accepting those falling within this threshold. Tuning the discrepancy measure and the parameter ***ɛ***, which trades accuracy for number of simulations, is difficult in practice and usually requires compressing the model output into low-dimensional summary statistics, a step that typically entails a loss of information.

A different simulator-based approach is synthetic likelihoods [29,30], where the likelihood is approximated by a multivariate Gaussian whose mean and covariance are estimated from simulations. We will refer to this method as Gaussian synthetic likelihoods (GSL). Like ABC, this approach frequently works with summary statistics of the data, which in this case should be approximately normally distributed under the model. In what follows, we will use the observed molecule numbers at different times. This approach is similar to the LNA, which models the reaction system as a linear set of stochastic differential equations and also results in a multivariate Gaussian distribution for observed molecule numbers. The difference is that this Gaussian is derived analytically under the LNA, while GSL estimates this Gaussian from simulations. The LNA is very cheap to evaluate and commonly used in inference [19,22,31], but for nonlinear systems, it provides biased estimates of the means and variances of molecule numbers and it is generally unable to model multimodal systems. Given enough simulations, GSL can be expected to be more accurate for those systems and will be therefore used as a comparison instead of the LNA.

For systems with highly non-Gaussian distributions, neither the GSL nor the LNA are likely to provide accurate results [32,33]: as shown in [24], Gaussian approximations can result in unusable parameter estimates for some systems. While parameters inferred using these methods will usually result in a good fit on the moment level, systems with non-Gaussian distributions are not uniquely defined by their means and variances, and there is no guarantee that the predicted parameters will match the shape of experimentally observed distributions. Methods that approximate the likelihood based on kernel density estimation [34] or neural networks [35] can better model non-Gaussian distributions, but they can require significant amounts of tuning and computational power to work well. A scalable approach to inference would ideally combine the flexibility of simulator-based methods with prior knowledge of the model to provide efficient yet flexible means of approximating the likelihood function.

In this paper, we propose a new method for inference in a wide class of biochemical reaction networks, specifically those modelling gene expression, which is rooted in the specific characteristics exhibited by models of gene regulatory networks. Gene expression systems can often be thought of as systems switching between discrete states of expression, broadly speaking corresponding to patterns of activation states of the genes’ promoters [36–39]. It is therefore natural to abstract the dynamics of gene systems as an indirectly observed dynamical system over a discrete (finite) set of states. These states are measured through observations of molecular counts; motivated by experimental measurements of the distributions of transcript and protein numbers, as well as analytical solutions of the CME obtained in a variety of cases, we propose a negative binomial mixture distribution as a model for molecular counts in our coarse-grained models of gene expression. We therefore propose that mixtures of time-dependent negative binomials can provide a tractable class of approximate models of gene expression systems. We call this class of models *synthetic models* (SM), and use these to approximate the likelihood function of gene expression models by fitting them to model simulations, in the spirit of synthetic likelihoods [30]. In cases when measurements are taken at short time intervals, where time correlations are particularly important, SM can be further enhanced by imposing hidden Markov model (HMM) dynamics on the latent states. We show that SM can provide excellent estimates of the model likelihood where other methods (FSP, GSL, MBI and ABC) struggle, and that our approach can be applied to obtain accurate parameter and uncertainty estimates for challenging inference problems.

### 1.1. Synthetic models

**Figure 1.**
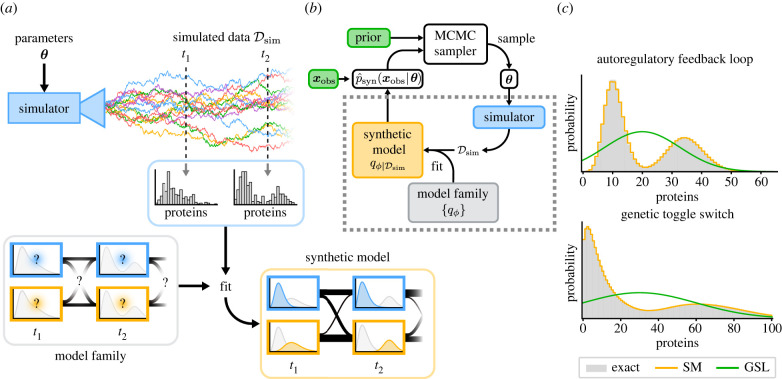
Bayesian inference using synthetic models. (*a*) Given a parameter set ***θ***, stochastic simulations of the original model are run to obtain samples $D_{\text{sim}}$. The synthetic model is picked from a parametric family of candidates and fit to the simulated samples. Our synthetic model is a finite-state Markov model with negative binomial output distributions for each state. (*b*) Synthetic models (dashed box) provide an approximation to the likelihood ${\hat{\;p}}_{\text{syn}}(x_{\mathrm{obs}}\;|\;\theta )$ that can be plugged into standard MCMC algorithms for parameter inference and Bayesian model selection. (*c*) Synthetic models based on negative binomial output distributions typically provide better fits to data than Gaussian approximations.

Our approach to inference is based on approximating the distributions predicted by the CME within a suitable family of candidates. We are in particular interested in gene expression systems, including those with feedback, but our methodology is general and can be applied to a large class of models, including non-Markovian models such as those recently considered in [40,41]. An outline summarizing the method can be found in figure 1*a*,*b*.

Theoretical investigations have shown that single-time marginal distributions predicted by the CME for a variety of models describing many of the major biomolecular processes affecting gene expression (transcription, translation, cell growth, DNA replication and cell division) can be approximated by mixtures of negative binomials (MNBs) in the presence of time-scale separation [42–46]—for an illustration see figure 1*c*. When time-scale separation is not applicable, such an approximation cannot be derived analytically, yet measurements of the distribution of mRNA and protein numbers in bacterial, yeast and mammalian cells show that these are still well fit by such mixtures in many cases [36–39].

Having established that MNBs provide a good statistical model for experimental measurements of gene expression networks at fixed times, the next step is to extend this to include time dependency. Experimental measurements of a system at different times will be correlated, and a natural way to emulate these correlations is to treat the individual mixture components at each time point as states in an HMM. More precisely, we propose using a finite-state Markov chain with negative binomial output distributions for each state, see figure 1*a* for an illustration. This statistically tractable surrogate model, which we term synthetic model, defines a surrogate distribution over observations jointly at all measured time points. Note that integrating out the hidden state variable shows that the marginal distribution at any time point is still a mixture of negative binomials.

Assuming the marginal distribution *p*(**x**|***θ***, *t*) predicted by the CME at time *t* can be approximated by a mixture of negative binomials *q*_*ϕ*_(**x**) with parameters *ϕ*, a principled way to determine these parameters is to minimize the Kullback–Leibler divergence between the two distributions

$$\phi^{*}=\underset{\phi }{\arg\;\min}D_{\mathrm{KL}}(p(\cdot |\theta ,t)\|\;q_{\phi }).$$

Here, *q*_*ϕ*_ is the MNB with mixture parameters *ϕ*. Since the reference distribution, being given by the solution of the CME is in general inaccessible, we can approximate it empirically by drawing samples using the SSA; minimizing the above KL divergence is then equivalent to maximizing the likelihood of the simulated samples, up to sampling error.

Fitting MNBs to data can be done efficiently using the expectation-maximization (EM) algorithm described in [47,48]. In order to fit all parameters of an HMM, including the initial distribution and the transition rates, we used the Baum–Welch algorithm (see electronic supplementary material for details), a special case of the EM algorithm that performs maximum-likelihood fitting for HMMs. Once we have fit our synthetic model, we can then compute the likelihood of our experimental observations **x**_obs_ using the forward algorithm for HMMs. This likelihood, which we denote ${\hat{\;p}}_{\text{syn}}(x_{\mathrm{obs}}\;|\;\theta )$, can be used to compute the posterior over parameters ***θ***, typically using MCMC, or to find the most likely parameters via optimisation—for an illustration see figure 1*b*. In most contexts, the observed data ***x***_obs_ will consist of independent and identically distributed measurements for many cells, and the synthetic model (or Gaussian for GSL) is used to evaluate the likelihood for each observation independently.

Our procedure to estimate the likelihood *p*(**x**_obs_|***θ***) for model parameters ***θ*** is as follows:

(1) Simulate sample trajectories using the SSA for the original model with parameters ***θ***.
(2) Fit the parameters of the HMM to the simulated trajectories using the Baum–Welch algorithm.
(3) Evaluate the HMM at the observed data ***x***_obs_ to obtain the synthetic likelihood ${\hat{\;p}}_{\text{syn}}(x_{\mathrm{obs}}\;|\;\theta )$.

Note that the synthetic model has to be fit from scratch for every parameter set at which the likelihood is queried, which is the main computational bottleneck of our approach.

The number of simulations and mixture components should be chosen appropriately for the reaction network. In our experiments, we simulated each system at several randomly chosen parameters and ensured that the given number of simulations and mixture components could accurately reproduce the observed distributions. In an MCMC context, the number of simulations should be chosen such that the variance of the likelihood estimate still results in an acceptable rejection rate. Allowing a few more components than necessary did not affect the quality of fit in our experiments, as extraneous components either merged with others or were assigned negligible weights.

We remark that experimental data for mRNA or protein number generally comes in the form of either population snapshot data or live cell imaging. In the case of the former, each snapshot represents a different group of cells and modelling correlations at different times becomes unnecessary; it therefore suffices to fit MNBs independently for each time at which a snapshot is taken. This simplification can also be made when time correlations are weak enough to be neglected, as is the case for the toggle switch model considered in the next section.

## 2. Results

### 2.1. Autoregulatory genetic feedback loop

**Figure 2.**
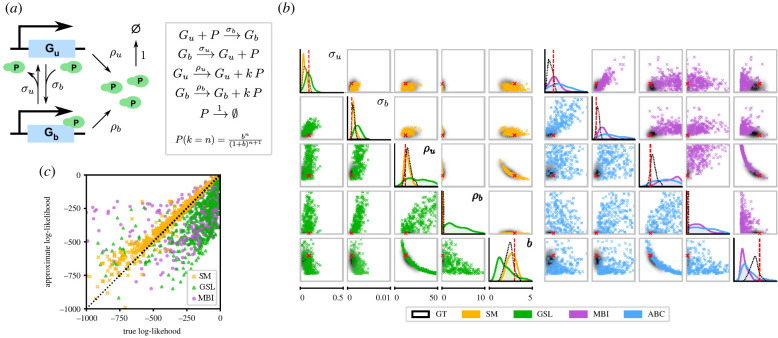
Comparison of synthetic models with standard methods for the case of an autoregulatory genetic negative feedback loop. (*a*) Illustration of the reaction scheme describing the genetic circuit. (*b*) Posteriors obtained using four different inference methods, with the ground truth solution computed using the FSP (black). The red dashed lines show the true parameter values. Left: synthetic models (SM) and Gaussian synthetic likelihoods (GSL). Right: moment-based inference (MBI) and sequential ABC. The ranges plotted coincide with the prior ranges. (*c*) Comparison of true and approximate log-likelihoods. Parameter values were sampled from the prior, and the true log-likelihoods were computed using the FSP. Synthetic models (yellow) provide significantly closer approximations to the true log-likelihood than either Gaussian synthetic likelihoods (green) or moment-based likelihoods (purple). The true parameter values are given in electronic supplementary material, figure S1. The input data consist of protein numbers from 25 SSA trajectories measured at times *t* = 4, 8, 12, 16.

We consider an autoregulatory genetic feedback loop that is illustrated in figure 2*a*. It consists of a gene with two promoter states G_u_ and G_b_, and a protein P that is produced at different rates *ρ*_*u*_ and *ρ*_*b*_ depending on the promoter state. Protein production occurs in geometrically distributed bursts with mean burst size *b*. The promoter switches from state G_u_ to G_b_ by binding a protein molecule with rate *σ*_*b*_, and this process is reversible with rate *σ*_*u*_. Protein dilution is effectively modelled by a first-order reaction; note that all other rates are rescaled by the protein dilution rate. We assume mass action kinetics for all reactions. This is the prototypical example of stochastic self-regulation in a gene and can be rigorously derived from a more detailed model incorporating mRNA dynamics [46].

We consider the negative feedback regime where the protein production rate decreases upon protein binding (i.e. *ρ*_*b*_ < *ρ*_*u*_). Due to the simplicity of this model, likelihoods can be efficiently computed using the FSP using a truncation to several hundred states in our examples, leading to an essentially exact solution and enabling us to compare our method with exact Bayesian inference. We tested our approach by using the SSA to simulate time-series data from several genetically identical cells and performed Bayesian inference based on the observed protein numbers, with a uniform box prior on the model parameters. For all methods except ABC, we sampled from the posterior using the Metropolis–Hastings sampler with a fixed Gaussian transition kernel (see electronic supplementary material, §1 for details). We note that while the steady-state solution of the CME of this system is predicted by theory to be well approximated by a negative binomial mixture (because of the small promoter switching rates compared with other rates [46]), we use data collected in pre-steady state where theoretical results are difficult to obtain. Hence the use of SM as a means to automatically obtain a negative binomial mixture approximation of the likelihood is particularly useful in this case.

Due to the presence of bimolecular protein–gene interactions, solving the moment equations for MBI in this model requires a moment-closure approximation. We used the linear mapping approximation (LMA) [49] for this purpose, which provided very accurate moment estimates for the parameter ranges considered in our experiments.

We compared the exact posterior obtained using the FSP with those computed using SM and three representative inference methods: GSL, MBI and ABC (figure 2*b*)—see Material and methods and electronic supplementary material for details. For all parameters, the mode of the posterior computed using FSP or SM is close to the true parameter values; this is not the case for the other methods. In particular, our approach was the only one to yield a posterior where *ρ*_*b*_ was concentrated around the true value of zero, whereas the other methods yielded posterior means that were significantly non-zero, falsely suggesting leaky gene expression. This is an example of technical parameter non-identifiability, where a structurally identifiable parameter cannot be identified using a specific method. As we see in this case, using detailed distributional information can be valuable for discriminating between different modes of gene expression. Figure 2*c* shows that SM approximate the true likelihood of the model substantially better than both GSL and MBI, uniformly over the range of parameters considered (ABC does not yield explicit likelihood estimates). See electronic supplementary material, figure S2 for further data including the posterior and MLE predictive distributions obtained using these methods.

In electronic supplementary material, figures S2 and S3, we repeat the same analysis for a positive feedback loop where the protein production rate increases upon protein binding (*ρ*_*b*_ > *ρ*_*u*_). As for the negative feeback loop, we find that likelihood approximation and parameter inference using SM is significantly more accurate than using standard methods.

### 2.2. Genetic toggle switch

**Figure 3.**
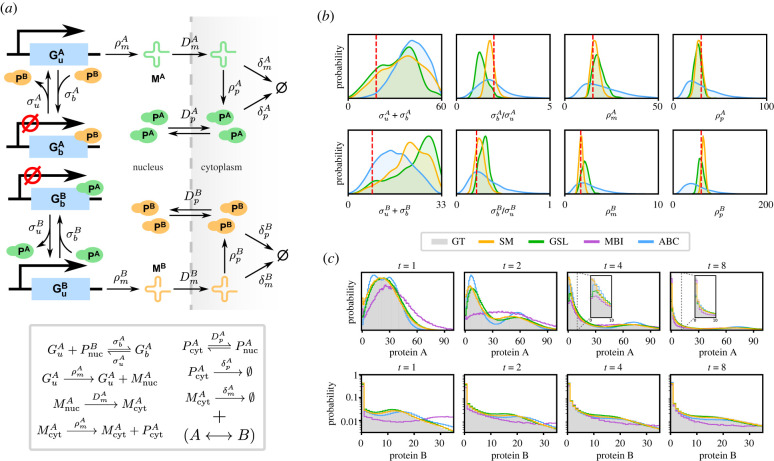
Comparison of synthetic models with standard methods for the case of a genetic toggle switch in a eukaryotic cell. (*a*) Illustration of the reaction scheme describing the circuit, which is symmetric in *A* and *B*. (*b*) Posteriors obtained using synthetic models, Gaussian synthetic likelihoods, moment-based inference and sequential ABC. (*c*) Predictive distributions generated using the SSA at four time points for the maximum-likelihood parameters obtained using each method. Synthetic models (yellow) and Gaussian synthetic likelihoods (green) provide significantly closer approximations to the true distribution (grey) than the other methods, with the former yielding a consistently more accurate fit for low molecule numbers (see insets). Parameters and prior ranges for all parameters are given in electronic supplementary material, figure S4. The input data consist of cytoplasmic protein numbers (A and B) from 100 SSA trajectories measured at times *t* = 1, 2, …, 8.

Next, we consider a genetic toggle switch [50] in a eukaryotic cell (for an illustration see figure 3*a*). This consists of two different promoters, each of which can be on or off, and the protein from each promoter represses the expression of the other. We explicitly model the translocation of mRNAs from the nucleus to the cytoplasm, the translation of cytoplasmic mRNAs into proteins and the translocation of proteins to the nucleus.

This system is significantly more complex than the autoregulatory feedback loop considered above, involving an effective 10 species (we do not count bound promoter states due to conservation laws). For realistic mRNA and protein abundances (few tens and several tens to hundreds, respectively), a simple state space truncation would need to consider of the order of 10^9^ states, many orders of magnitude more than the previous example. Due to hardware constraints, we were therefore not able to apply the FSP to this example; this illustrates the lack of scalability of the direct approach when applied to more realistic systems and the need for more efficient methods.

Fixing the translocation and degradation rates, which can often be deduced experimentally, we tested our approach in this case by inferring the remaining eight parameter values. We used the SSA to simulate a synthetic dataset of 100 cells observed at eight different time points each, and performed Bayesian inference on the cytoplasmic protein numbers (both species) with a box prior around the true parameters (figure 3*b*) using SM, Gaussian MBI (not shown, see below) and ABC. As with the autoregulatory feedback loop, we used a Metropolis–Hastings sampler with a Gaussian transition kernel for all methods except ABC (see electronic supplementary material for details).

Not all parameters of this model were identifiable from the data: while the ratio between the binding and unbinding rates for each gene can be identified, the individual rates themselves cannot. These findings did not depend on the method used, which suggests that we are dealing with structural parameter non-identifiability, as opposed to technical non-identifiability due to the shortcomings of an individual method. This is supported by electronic supplementary material, figure S4, which shows that the predictive uncertainty in the posteriors is very small despite large variations in these two parameters. By contrast, the peaked posteriors around the true values of the transcription and translation rates show that these rates can be well estimated by SM and GSL (which is not the case for ABC and MBI). We furthermore compared the predictive distributions for the maximum-likelihood parameters estimated during inference (figure 3*c*)—we note that the SM prediction is the only one of all methods that is accurate for all times.

While the input to this experiment consisted of time-series data for SM, fitting a full HMM performed similarly to fitting independent MNBs at each time point, and we therefore used the latter approach for simplicity. We observed that using a full HMM for this model was more prone to local optima during the fitting step, which resulted in a higher variance of the approximate likelihood and reduced acceptance rates. GSLs similarly had significantly lower acceptance rates compared with independent MNBs, with a correspondingly increased number of MCMC iterations until convergence.

As for the autoregulatory feedback due to the nonlinearity of the propensities of the protein–gene interactions, MBI for this model requires a moment-closure approximation. Out of the nine different schemes implemented in the package MomentClosure.jl [51], the LMA [23,49] was the only one that consistently predicted positive moments around the true parameters, a necessary condition to get well-defined likelihoods. However, even the LMA failed to predict the moments accurately for this system, resulting in a wildly skewed posterior (not shown) and heavily divergent predictive distribution (figure 3*c*).

### 2.3. MAPK pathway in *S. Cerevisiae*

**Figure 4.**
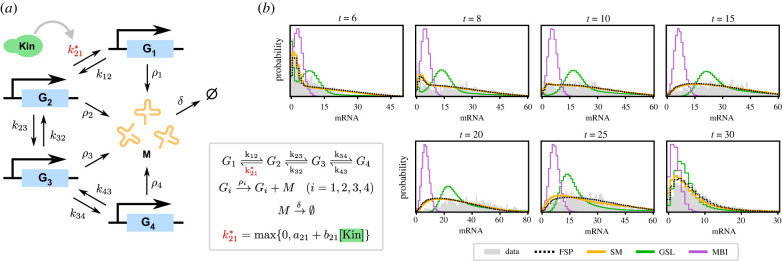
Comparison of synthetic models with standard methods for the MAPK pathway model. (*a*) Illustration of the reaction scheme of the model, which consists of a gene in four possible states *G*_*i*_ and mRNA. A kinase, whose concentration is a time-dependent input signal, modulates the transition rate $k_{21}^{*}$ (see electronic supplementary material for details). (*b*) Comparison of the experimentally observed distribution (grey) with the predictive distributions for the maximum likelihood estimates obtained using four different methods. Synthetic models (yellow) provide a quality of fit similar to the finite state projection (dotted line), whereas Gaussian synthetic likelihoods (green) and moment-based inference (purple) fail to capture the long-tailed shape of the distributions. Estimated parameters for each method are given in electronic supplementary material, table S1.

Our final example uses experimental data from [52] to analyse the high osmolarity glycerol MAPK pathway in *S. Cerevisiae*, where population snapshots were taken at different times after the induction of osmotic shock. The model is described in figure 4*a* which features highly non-Gaussian distributions of mRNA copy numbers. It consists of a single gene (*STL1*) in four possible states, each of which produces mRNA at a specified rate. Switching into one specified state is controlled by a kinase that is activated by a signalling cascade under osmotic shock; the concentration of the kinase is given as an external input to the system.

It was found in [24] that MBI generally fails to yield good predictive results for this example, in contrast to direct likelihood-based inference using the FSP. As the model contains only four effective species (mRNA and three gene states, the fourth being given by the conservation law) it is very amenable to the FSP, as a truncation to a few hundred states suffices to capture its dynamics in the relevant parameter range. Despite this simplicity, the model has 12 free parameters and poses a challenge for full Bayesian inference. A random-walk Metropolis–Hastings algorithm would require very small step sizes in order to keep acceptance rates high in 12 dimensions, requiring very long run-times in order to cover the relevant posterior mass. For synthetic likelihood-based approaches, another issue is the large number of experimental measurements (16 k), which significantly increases the variance of the total likelihood estimates and reduces acceptance rates even further. For these reasons, we followed the approach of the authors in [24,52], performing maximum-likelihood estimation (MLE) and comparing the predictive distribution with experimental data.^1^

The results can be seen in figure 4*b*. Since the data comes in the form of independent population snapshots, we used independent MNBs as our synthetic model. Parameters obtained using FSP and our approach provide good agreement with the experimental data in [24], whereas GSL and MBI failed to match the observed data. MBI was not able to accurately estimate the moments for this system, resulting in biased parameter estimates that did not agree with the inputs to any appreciable degree. GSL returned parameter estimates which predict distributions with means and variances that match the data, but with appreciably different shapes—this is an example of technical parameter non-identifiability, owing to the fact that GSL reduces the data to its first two moments. By contrast, MNBs model the data on the distribution level, and the parameters estimated using these provide a close match to the data. Our results show that synthetic models can be applied to obtain high-quality parameter estimates for real-life biochemical systems with comparable accuracy to FSP.

## 3. Discussion

We presented an approach for inference in stochastic gene regulatory networks relying on an approximation of the generally intractable CME by a family of SM, fit to the original model via simulations. These SM yield estimates of the model likelihood, which can be optimized to obtain MLE for the true model parameters, or within an MCMC sampler for posterior inference and model selection.

We tested our method on a well-studied autoregulatory feedback loop and showed that it closely approximates the exact posterior in both the positive and the negative feedback regimes, recovering true parameters with significantly more accuracy than standard approaches such as MBI and ABC, both in terms of the posterior approximation and in terms of the predicted model output. We then considered a more complex model, the genetic toggle switch, which is difficult to analyse using moment-based methods and the FSP, illustrating the flexibility of our approach and its ability to handle non-trivial models of real-life systems. We finally demonstrated the effectiveness of our approach for analysing real-life data by testing it on the MAPK pathway in *Saccharomyces cerevisiae* in [52], obtaining parameter estimates rivalling those of the FSP in predictive accuracy. Our findings show that distributional approximations beyond Gaussians can aid parameter identifiability, and that simulation-based methods can be effectively used in place of analytical approximations where the latter fail.

The main contribution in this work is an alternative simulation-based class of approximations to the CME. As inference for the CME generally relies on approximations, the chosen approach for a given reaction network can have a large impact on parameter inference. For small enough systems, the FSP can provide an excellent finite-dimensional approximation with practically negligible error. MBI, which replaces the full likelihood by that of empirical moments, can be accurate given large enough sample sizes, but it relies on the true moments being computable, which is not the case for general reaction networks with bimolecular reactions. Furthermore, the moments themselves do not always carry enough information to identify parameters uniquely, particularly for very non-Gaussian distributions; this is also a potential issue with ABC where informative summary statistics have to be chosen, but the appropriate choice is not clear *a priori*. This can lead to overly broad or otherwise inaccurate posteriors in practice, as we observed in our experiments.

Gaussian approximations such as the LNA and GSL are often very practicable and easy to implement, and they can perform very well if the true distributions are not far from Gaussian. Here, the bias introduced by the LNA to systems with bimolecular reactions contrasts with the variance involved in estimating the GSLs. As we observe in the genetic toggle switch, even for systems with markedly non-Gaussian likelihoods these methods can provide useful parameter estimates, but in general their inability to distinguish between distributions with a given mean and variance leads to potentially unreliable results. MNBs can provide very accurate approximations of the distributions occurring in many stochastic reaction networks, including very non-Gaussian ones, and one would expect their use to result in more accurately inferred parameters in general, as corroborated by the above experiments.

We emphasize, however, that more accurate SM than the HMMs introduced in this paper could be used especially for strong time correlations between measurements; for closely spaced observations leading to such correlations sequential Monte Carlo methods such as [53] are likely to provide better results. It should be remarked that the improvement in accuracy that can be obtained using our approach is probably not uniform in parameter space. Indeed, many configurations of parameters will yield species distributions which can be reasonably well approximated as Gaussians: in these cases, while we still expect our method to perform well, we do not expect it to differ significantly from GSL or MBI. It is worth noticing, however, that many biologically interesting phenomena arise precisely when systems are far from Gaussianity, for example exhibiting multi-modality.

A major limitation of our method is that fitting a synthetic model to simulations introduces a variance in the approximate likelihood proportional to the number of experimentally observed datapoints. In order to obtain accurate estimates of the true likelihood, therefore, the number of simulations used to train the synthetic model needs to be increased in step with the sample size. For MLE estimation, this does not significantly complicate things, but in an MCMC context this variance causes difficulties as it can heavily reduce acceptance rates. Another limitation of our method is that a MNB can only provide an accurate approximation for (transcript or protein) marginal distributions with a Fano factor greater than 1. This condition is met in the overwhelming majority of computational models and experimental studies of gene regulatory systems, but exceptions exist [54–56]. Incorporating a different parametric family of distributions with Fano factor smaller than 1 (e.g. hypergeometric) is in principle straightforward within the SM framework.

The Metropolis–Hastings sampler used in this paper is most suited for low-dimensional problems spaces, as a random walk-based approach is not an efficient way to explore high-dimensional posteriors. MCMC sampling in high-dimensional spaces is often done using the Metropolis-adjusted Langevin algorithm or Hamiltonian Monte Carlo [57], both of which require gradients of the posterior to direct the sampler towards high-probability regions. Approximating the gradient of the likelihood function using synthetic likelihoods is therefore a promising direction for future research.

The need to fit a synthetic model from scratch at every iteration of the MCMC procedure is the main computational bottleneck of our method. Methods such as data subsampling [58,59] and amortization, e.g. using neural networks [35], could result in significant speed-ups and a reduced variance in the likelihood estimates for more complex problems.

An advantage of our approach over standard CME-based inference methods is that it can be readily applied to systems with extrinsic noise, simulated using the exact Extrande algorithm [60], and/or non-Markovian systems such as those considered in [40,41,61,62]. While such models are difficult to analyse mathematically, requiring various extensions to the CME formalism, the presence of efficient and exact versions of the SSA for these systems allows most simulator-based inference methods to work without any modification. We hope that our work, as well as the ideas contained within, provides a useful stepping stone that will enable researchers to analyse and use these models more efficiently in the future.

## 4. Material and methods

### 4.1. Gaussian synthetic likelihoods

We model the distribution of observed molecule numbers, jointly at all time points, as a multivariate Gaussian whose mean and covariance we estimate from simulations obtained using the SSA. If *S* species are simultaneously observed at *T* time points, this results in a *S* × *T*-dimensional Gaussian which is fit to the simulations by MLE. We then evaluated the likelihood for each observed cell using this inferred Gaussian.

### 4.2. Moment-based inference

The moments of the CME can be computed at any point in time from its associated moment equations. For linear systems with mass action kinetics, such as the MAPK pathway example, these can be solved directly, while for the autoregulatory feedback loop and the genetic feedback loop we used the LMA [49] to obtain a solvable set of equations.

Experimentally observed moments form a stochastic estimate of the true moments of the system; for large sample sizes, the central limit theorem ensures that these sample moments will be approximately normally distributed. Following the approach in [17,23] we thus model the first and second (uncentred) sample moments over observed molecule numbers using a multivariate Gaussian. The means and covariances of the sample moments can be expressed in terms of the analytical moments of the system, and we assume that measurements at different time points are independent (see electronic supplementary material for details). This results in a Gaussian likelihood on the moment level that can be used for inference. If *S* species are observed, for each time point, the associated Gaussian will have *S* components for the means and *S*(*S* + 1)/2 components for the second moments. We estimate the likelihood of the observed data by evaluating the likelihood of the empirical first and second moments using this Gaussian.

### 4.3. Approximate Bayesian computation

We use the first- and second-order moments over species numbers at each time point as summary statistics. Fixing a tolerance ***ɛ***, we repeatedly sample parameters from the prior and compare the simulator output ***x*** with the observed data ***x***_obs_. Namely, we accept parameters for which the sum of the squared relative errors in the first and second moments is less than ***ɛ*** and iterate until a pre-specified number of acceptances is reached. To improve sample efficiency, we decrease ***ɛ*** over multiple rounds following [28], using a Gaussian proposal prior estimated from the results of the previous round to guide sampling. Regression adjustment [63] did not yield measurable improvements in our experiments.

## Data Availability

Code implementing synthetic models as well as the experiments in this paper is available at https://github.com/kaandocal/synmod. The data are provided in the electronic supplementary material [64].

## Endnote

1 We emphasize that the predictive distributions are obtained by running the model at the estimated parameters and are *not* MNBs (for SM) or Gaussians (for GSL).
